# Teriflunomide Treatment of Multiple Sclerosis Selectively Modulates CD8 Memory T Cells

**DOI:** 10.3389/fimmu.2021.730342

**Published:** 2021-10-05

**Authors:** Gaëlle Tilly, Marion Cadoux, Alexandra Garcia, Jérémy Morille, Sandrine Wiertlewski, Claire Pecqueur, Sophie Brouard, David Laplaud, Nicolas Degauque

**Affiliations:** ^1^Université de Nantes, CHU Nantes, Inserm, Centre de Recherche en Transplantation et Immunologie, UMR 1064, ITUN, Nantes, France; ^2^CHU Nantes, Service de Neurologie, CRC-SEP, CIC1413, Nantes, France; ^3^Université de Nantes, CRCINA, INSERM, CNRS, Nantes, France

**Keywords:** multiple sclerosis, teriflunomide, CD8 T cells, CD4 T cells, migration, memory T cells

## Abstract

**Background and Objectives:**

Inhibition of *de novo* pyrimidine synthesis in proliferating T and B lymphocytes by teriflunomide, a pharmacological inhibitor of dihydroorotate dehydrogenase (DHODH), has been shown to be an effective therapy to treat patients with MS in placebo-controlled phase 3 trials. Nevertheless, the underlying mechanism contributing to the efficacy of DHODH inhibition has been only partially elucidated. Here, we aimed to determine the impact of teriflunomide on the immune compartment in a longitudinal high-dimensional follow-up of patients with relapse-remitting MS (RRMS) treated with teriflunomide.

**Methods:**

High-dimensional spectral flow cytometry was used to analyze the phenotype and the function of innate and adaptive immune system of patients with RRMS before and 12 months after teriflunomide treatment. In addition, we assessed the impact of teriflunomide on the migration of memory CD8 T cells in patients with RRMS, and we defined patient immune metabolic profiles.

**Results:**

We found that 12 months of treatment with teriflunomide in patients with RRMS does not affect the B cell or CD4 T cell compartments, including regulatory T_REG_ follicular helper T_FH_ cell and helper T_H_ cell subsets. In contrast, we observed a specific impact of teriflunomide on the CD8 T cell compartment, which was characterized by decreased homeostatic proliferation and reduced production of TNFα and IFNγ. Furthermore, we showed that DHODH inhibition also had a negative impact on the migratory velocity of memory CD8 T cells in patients with RRMS. Finally, we showed that the susceptibility of memory CD8 T cells to DHODH inhibition was not related to impaired metabolism.

**Discussion:**

Overall, these findings demonstrate that the clinical efficacy of teriflunomide results partially in the specific susceptibility of memory CD8 T cells to DHODH inhibition in patients with RRMS and strengthens active roles for these T cells in the pathophysiological process of MS.

## Introduction

The selective and reversible inhibition of dihydroorotate dehydrogenase (DHODH), a key mitochondrial enzyme in the *de novo* pyrimidine synthesis pathway, by teriflunomide has been shown to be a safe and effective strategy to treat patients with multiple sclerosis (MS). The results obtained from phase 3 clinical trials demonstrated that patients with MS treated with teriflunomide show a significant reduction in disability progression and relapse rate and lower magnetic resonance imaging evidence of disease activity (1, 2). The primary mechanism of action explaining the success of DHODH inhibition is the suppression of stimulated T and B cell proliferation (3–5) by inducing metabolic stress in metabolically proliferating lymphocytes. The addition of exogenous uridine can overcome the antiproliferative effect of teriflunomide (6), and the selective effect on activated T and B cells is partially explained by the activation of the salvage pathway in resting cells to meet their pyrimidine needs (7, 8). Nevertheless, the mechanism of immune system modulation induced by teriflunomide remains unclear, with contradictory results reported. A decrease in only the frequency of CD45RA^+^ effector memory (TEMRA) CD4 T cells and plasmablasts has been reported for patients with MS after 6 months of treatment, while the frequency of other immune cells, including CD8 T cells, remained similar before and after DHODH inhibition (9). The production of proinflammatory cytokines (IFNγ, TNFα and IL-17) by CD4 T cells was not modified after 6 months of treatment (9). In contrast, another study reported a specific decrease in IFNγ, TNFα and IL-17 in both the CD4 T_H_1 and CD8 T cell compartments (10). High-affinity CD4 and CD8 T cell clones have been shown to be more susceptible to DHODH inhibition, and individual T cell clones are eliminated from the TCR Vβ repertoire of patients with MS within weeks of initial teriflunomide treatment (10). The contradiction regarding the impact of DHODH inhibition on the immune system has prompted the need to further characterize the immune system of MS patients, particularly with a longitudinal paired analysis and high-dimensional approach.

Immunopathology of MS has been widely studied, and based on models of experimental autoimmune encephalomyelitis, the concept of MS as an autoimmune disease mediated by CD4 T_H_1/T_H_17 cells specific to autoantigens of the CNS (11) has emerged. As major attention has been given to the CD4 T cell compartment in the analysis of MS patients, the impact of DHODH inhibition on CD8 T cells has not been carefully investigated. Nevertheless, our results and other accumulated evidence suggest a major role of CD8 T cells. Active lesions are infiltrated mainly by CD8 T cells that are in close proximity to oligodendrocytes and demyelinating axons (12, 13). CD8 T cell clones are shared between CSF and active white matter lesions (14, 15) and are also present in peripheral blood (15). Moreover, the involvement of DHODH in the key process of CD8 T cell migration has not yet been investigated, although the recirculation of memory T cells is a central process in the establishment of the immune response involving the localization of memory CD8 T cells in secondary lymphoid organs and nonlymphoid tissues.

In this study, we investigated the impact of DHODH inhibition in patients with relapse-remitting MS (RRMS) before and 12 months after treatment using high-dimensional flow cytometry to characterize the immune system as a whole. We demonstrated that DHODH inhibition had no impact on the CD4 T compartment without any modification in the frequency of CD4 T helper cells, CD4 T follicular cells or CD4 regulatory T cells or in the function of CD4 T helper and CD4 regulatory cells. In contrast, we showed that DHODH inhibition impacted the proliferation of CD8 T cells and the production of proinflammatory cytokines through effector memory (EM) and TEMRA CD8 T cells and their migratory properties. Finally, we demonstrated that the susceptibility of memory CD8 T cells to DHODH inhibition was not related to impaired metabolism.

## Methods

### Standard Protocol Approvals, Registrations, and Patient Consents

Patients included in the study suffered all from relapsing-remitting MS as defined by the revised MacDonald criteria published in 2005, and the demographic and clinical characteristics are shown in **Table 1**. Twelve out of 15 MS patients were naïve of any treatment at the time of teriflunomide start. The three other patients were previously treated with an immunomodulatory treatment (Interferon beta, dimethyl-fumarate) that was stopped at least three months before Teriflunomide initiation. Finally, Teriflunomide was started at least 6 months after a relapse for all the patients. The University Hospital Committee and the Committee for the Protection of Patients from Biological Risks approved the study (ABM PFS13-003 “Collection sclérose en plaques”), and all patients were recruited after obtaining informed signed consent.

**Table 1:** Demographical and clinical characteristics of the patients at sampling.

Form of the disease (%)	Sex ratio	Disease duration [years, median (IQR_25-75%_)]	Time between last flare and treatment initiation	Age [years, median (IQR_25-75%_)]	EDSS [median (IQR_25-75%_)]	Sampling at flare	Previous treatment
			[months, median (IQR_25-75%_)]			(Y/N; n)	(Y/N; n)
**RR 100%**	5 M/10 W	3.00	30	39.00	0.50	13/2	12/15
		[0.17 – 17.00]	[15 – 48]	[31.00 – 44.00]	[0.00 – 1.25]		

### Blood Samples

PBMCs were separated from blood samples collected in EDTA tubes on a Ficoll gradient layer according to the manufacturer’s recommendations and were frozen in autologous serum with 10% DMSO. PBMCs were then thawed in CTL wash supplemented with benzonase (10 U/mL).

### Reagents

The antibodies used for flow cytometry analyses and key reagents are listed in **Supplementary Tables 1** and **2**.

### CD8 T Cell Isolation and FACS-Sorting of CD8 T Cell Subsets

PBMCs were cultured overnight in complete RPMI medium supplemented with 10% FCS, 100 U/mL penicillin and 100 U/mL streptomycin. CD8 T cells were purified using a human REAlease CD8 MicroBead kit. Naïve (CD45RA^+^CCR7^+^), EM (CD45RA^-^CCR7^-^) and TEMRA (CD45RA^+^CCR7^-^) CD3^+^CD8^+^ cells were sorted from PBMCs by FACS (FACS Aria, BD Biosciences; the purity was greater than 95%).

### Immune Profiling by Spectral Flow Cytometry

Two million PBMCs were either directly stained with the “T cell panel” or stimulated with PMA (50 ng/mL) and calcium ionophore (500 ng/mL) for 3.5 h in the presence of brefeldin A (5 µg/mL) before being stained with the “functional panel” (i.e., IL-10, IL-13, IL-17, IFNγ, TNFα). PBMCs were analyzed using an Aurora 5-laser spectral flow cytometer (Cytek). After individual titration of each antibody, a deconvolution matrix was set up according to Cytek recommendations. Quality control procedure was performed prior each data acquisition according to Cytek recommendations.

### High-Dimensional Data Analysis of Flow Cytometry Data

Supervised and unsupervised (optSNE and FlowSOM) analyses were performed on OMIQ (https://www.omiq.ai/). FCS files were first cleaned using FlowCut (16) and by gating on CD45^+^Singlet^+^LiveDead^-^. Non-naïve CD4 T cells and non-naïve CD8 T cells were analyzed separately. optSNE analysis was performed using equal amounts of samples comprising 1x10e5 non-naïve CD4 T cells or 5x10e4 non-naïve CD8 T cells obtained from each FCS pool, with 1000 iterations, a perplexity of 30, and a theta value of 0.5. For the non-naïve CD4 T cells, the expression of the following markers was measured CCR6, CXCR3, CCR4, CD161, CXCR5 and CD28. For the non-naïve CD8 T cells, the expression of the following markers was measured: CCR6, CXCR3, CCR4, CD161, CXCR5 and CD28. For non-naïve CD8 T cells, the expression of the following markers was measured: CD161, CD69, CXCR3, CD57, CD45RA, CD152, CXCR5, CD137, PD-1, TIGIT, CD39, CD194, CCR7, CD49a, TIM3, CD27, LAG3, CD28, KI-67, CD25, CD127, CD73 and CCR6. The resulting optSNE maps were fed into the FlowSOM clustering algorithm (17). For each cell subset, a new self-organizing map (SOM) was generated using hierarchical consensus clustering on the tSNE axes. For each SOM, 12 and 20 metaclusters were identified for non-naïve CD4 T cells and for non-naïve CD8 T cells, respectively.

### Metabolic Assays

After FACS sorting, purified CD8 T cell subsets were cultured overnight in TexMACS buffer at 37°C and 5% CO2. The oxygen consumption rate (OCR) and extracellular acidification rate (ECAR) were measured using 4×10^5^ or 2.5×10^5^ purified CD8 T cell subsets and Seahorse XF24 or XF96 analyzers, respectively. The assay was performed in Seahorse XF-base medium supplemented with 10 mM glucose, 2 mM glutamine, and 1 mM pyruvate. A mitochondrial stress assay was performed by successively adding oligomycin (1.5 μM), CCCP (1 μM), and anti-mycin A + rotenone (1 μM each). To assess the OCR and ECAR upon polyclonal stimulation, PMA (50 ng/mL) and ionomycin (500 ng/mL) were added 75′ after the start of the experiment. 2-DG (250 mM) was added before stimulation with PMA-Iono.

### Quantification of Mitochondrial and Mitochondrial Membrane Potential (MMP)

MitoTracker green and MitoTracker red were used to quantify mitochondria and to assess the MMP, respectively. PBMCs were first incubated with 100 nM MitoTracker green or MitoTracker red for 30′ at 37°C and 5% CO2, and then, the cells were stained using anti-CD3, anti-CD8, anti-CD45RA, and anti-CD28 antibodies. The samples were immediately analyzed with an LSRII flow cytometer.

### Glucose Transporter Expression

PBMCs were stimulated overnight or not with aCD3 (2 µg/mL) and then stained using anti-CD3, anti-CD8, anti-CD45RA, anti-CD28 and anti-Glut1 antibodies according to the manufacturer’s instructions. GLUT1 expression in CD8 T cell subsets was assessed according to mean fluorescence intensity (MFI).

### Activation and Proliferation

CD8 T cell isolation or FACS-sorted CD8 T cell subsets were plated in 96-U bottom plates coated with anti-CD3 mAb (2 μg/mL) and cultured for 1–5 days in RPMI complete medium with IL-15 (10 ng/mL) or anti-CD28 mAb (2 µg/mL). Proliferation was monitored using diluted cell proliferation dye eFluor450 (CPD) and assessed according to the frequency of CPD^low^ cells on days 4 and 6. After culturing overnight, activation (CD25 and CD69) and proinflammatory cytokine production (TNFα and IFNγ) of the CD8 T cell subset was determined by cytometry. Teriflunomide (100 µM), uridine (50 µM) and 2-DG (10 mM) were added as indicated.

### Cell Migration Assessment Using Time Lapse Microscopy

TEMRA (CD45RA^+^CCR7^-^) and EM (CD45RA^-^CCR7^-^) CD3^+^CD8^+^ cells obtained from MS patients were sorted by FACS and cultured overnight in complete RPMI medium supplemented with IL-15 (2 ng/ml). CD8 T cell subsets stained with the cytosolic Ca^2+^ indicator Fura-2 AM probe (1 µg/mL for 45 min at room temperature) were seeded on a confluent monolayer of primary Human Dermal Microvascular Endothelial [HDMECs that have been previously and widely used as a good model for the *in vitro* study of inflammatory responses (18)] activated for 24 h with TNFα (100 U/ml) in µ-Slide 8 wells. Teriflunomide (100 µM) or DMSO (negative control) was added after 20 min of cell migration. Cell migration was tracked by time-lapse microscopy through a 20× objective of a Leica DMI 6000B microscope equipped with MetaMorph® software (Molecular Devices), and 120 sequential images were captured in 20-second intervals. The migration paths of individual cells were determined with ImageJ software, TrackMate-extras plugin and in-house scripts (R Studio), and these data were used to calculate average migratory velocity according to the definition of Meyjering (19) during the observation period.

### Statistical Analysis

All statistical analyses were performed using GraphPad Prism. Mann-Whitney U tests, Kruskal-Wallis tests followed by Dunn’s *post hoc* tests, and paired Wilcoxon tests were used as appropriate; the test performed is noted in the figure legends. All p-values are presented as exact values with * indicated as follows: * p<0.05; ** p<0.01; *** p<0.001; and **** p<0.0001.

### Data Availability

The data that support the findings of this study are available from the corresponding author upon reasonable request.

## Results

### In-Depth Characterization of CD4 T Cell Subsets in Teriflunomide-Treated Patients With RRMS Shows No Impact After DHODH Inhibition

**Figure 1:**
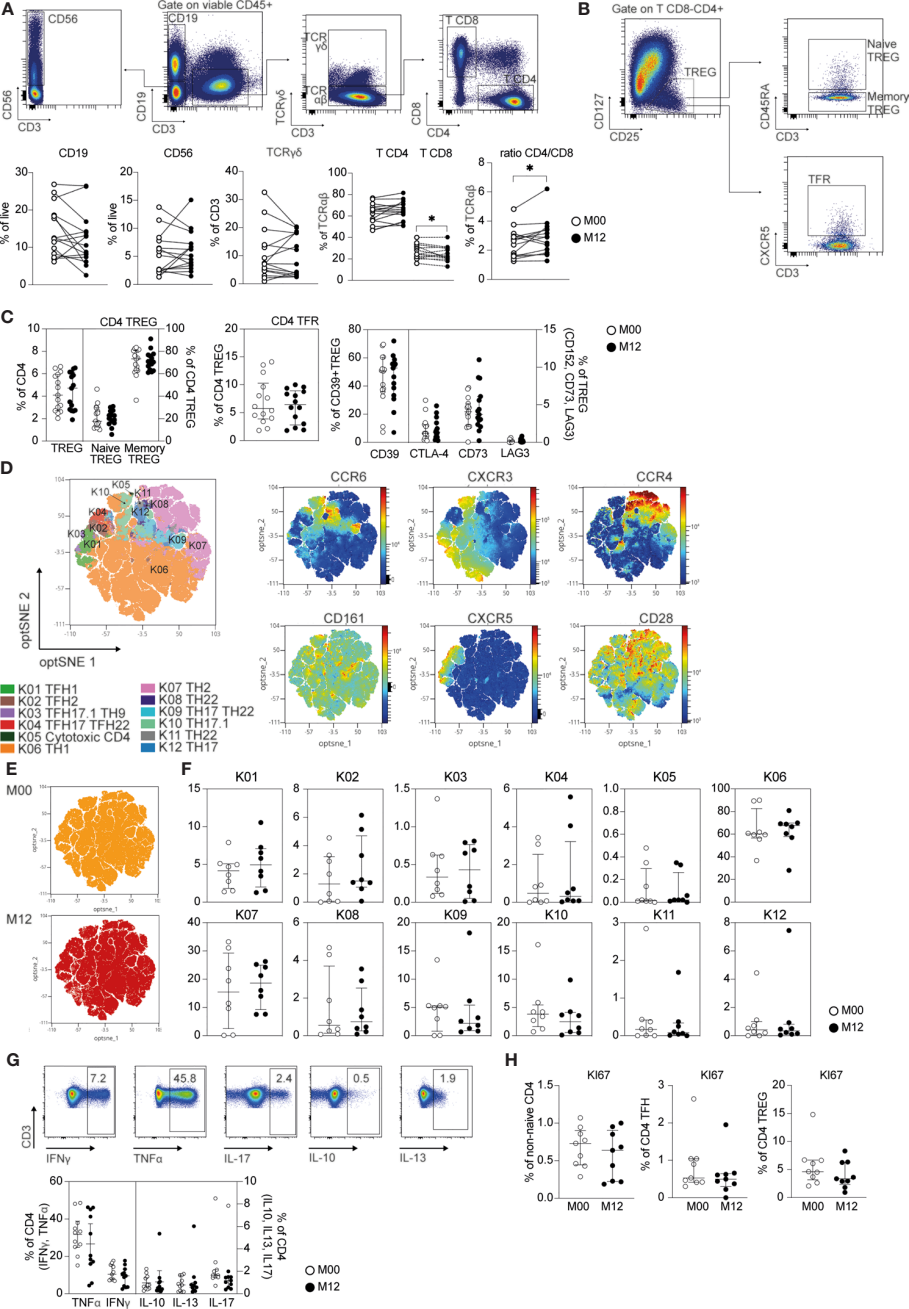
CD4 and CD8 T cell changes in teriflunomide-treated patients with RRMS. **(A)** Main immune cell populations in patients with RRMS at baseline (white) or 12 months post teriflunomide treatment (black; n=15). The gating strategy is shown after exclusion of dead cells, doublets and CD45^-^ cells (B cells, CD19^+^CD3^-^CD56^-^; NK, CD56^+^CD19^-^CD3^-^; γδ T cells, CD19^-^CD56^-^CD3^+^TCRγδ^+^; αβ CD4 T cells, CD19^-^CD56^-^CD3^+^TCRγδ^-^CD8^-^CD4^+^; αβ CD8 T cells, CD19^-^CD56^-^CD3^+^TCRγδ^-^CD8^+^CD4^-^). **(B, C)** Frequency of CD4 T_REG_ and CD4 T_REG_ subsets in patients with RRMS at baseline (white) or 12 months post-teriflunomide treatment (black; n=15). The gating strategy is shown after initial gating on CD19^-^CD56^-^CD3^+^TCRγδ^-^CD8^-^CD4^+^ (T_REGS_, CD25^high^CD127^low^; naïve T_REGS_, CD25^high^CD127^low^CD45RA^+^; memory T_REGS_, CD25^high^CD127^low^CD45RA^-^; T_FRS_, CD25^high^CD127^low^CXCR5^+^). **(D)** optSNE projection of non-naïve αβ CD4 T cell clusters identified by FlowSOM metaclustering. optSNE projections of the expression of the indicated proteins are shown. **(E)** Global optSNE projection of non-naïve αβ CD4 T cells for all participants pooled, with non-naïve αβ CD4 T cells of patients with RRMS at M00 and M12 post-teriflunomide treatment concatenated and overlaid. **(F)** Percentage of non-naïve αβ CD4 T cells from each timepoint in each FlowSOM cluster. **(G)** T_H_1 cell (IFNγ, TNFα), T_H_2 cell (IL-4, IL-13), T_REG_ (Il-10) and T_H_17 cell (IL-17) cytokine production by CD4 T cells in patients with RRMS at M00 (white) and M12 post-teriflunomide treatment (black) after polyclonal restimulation. **(H)** KI67 expression in non-naïve αβ CD4 T cells, T_FH_ cells (non-naïve αβ CD4 CXCR5^+^) and CD4 T_REGS_. **(A, C, E–H)** Each dot represents a unique patient, and the median and interquartile ranges (IQRs) are shown. Significance was determined by unpaired Mann-Whitney U or paired Wilcoxon test. *p < 0.05.

Using high-dimensional flow cytometry, we investigated the impact of DHODH inhibition in patients with RRMS before and 12 months posttreatment. The clinical characteristics of the patients are depicted in **Table 1**. We first focused on major lymphocyte populations (**Figure 1A**). The frequencies of leukocytes among B lymphocytes, NK CD56 cells, TCRγδ T cells and TCRαβ CD4 T cells were similar before and after teriflunomide treatment. Interestingly, a significant decrease in TCRαβ CD8 T cells was observed in patients with RRMS 12 months posttreatment (**Figure 1A**), resulting in an increase in the ratio of CD4/CD8 TCRαβ T cells (**Figure 1A**). We then applied high-dimensional flow cytometric analysis to investigate the CD4 T cell subsets. The CD4 T cell regulatory compartment was not impacted by DHODH inhibition, as shown by the similar frequency at baseline and 12 months posttreatment of CD4 regulatory T cells (T_REGS_; CD25^high^CD127^low^), memory CD45RA^-^CD4 T_REGS_, naïve CD45RA^+^CD4 T_REGS_ and follicular CXCR5^+^CD4 T_REGS_ (**Figures 1B, C**). The expression of functional markers of CD4 T_REGS_ was also not impacted by DHODH inhibition, as the expression of regulatory associated molecules by CD4 T_REG_, such as CTLA-4, LAG-3, and ectonucleotidases CD39 and CD73 CD4 T_REG_ (20), was similar before and 12 months post treatment (**Figure 1C**). To gain more insights into the T helper (T_H_) and T follicular helper (T_FH_) CD4 T cells, we applied global high-dimensional mapping of markers (CCR6, CXCR3, CCR4, CXCR5, CD161 and CD28) related to T_FH_, T_H1_, T_H2_, T_H17_, T_H17.1_, T_H22_ T_H9_ and cytotoxic CD4 T cells [identified as described in a consensus paper (21)]. An optimized t-distributed stochastic neighbor embedding (opt-SNE) representation (22) of the data highlighted 12 major populations that were identified by FlowSOM clustering and the comparison of marker expression (**Figure 1D**). The opt-SNE representation of non-naïve CD4 T cells of patients at M00 was similar to that of patients 12 months posttreatment (**Figure 1E**) and was also evidenced by the analysis of the frequency of T_H_ and T_FH_ CD4 T cell clusters (**Figure 1F**). The cytokine profiles (T_H_1, IFNγ, TNFα; T_H_2, IL-13; T_H_17, IL-17; and T_REGS_, IL-10) produced by TCRαβ CD4 T cells (**Figure 1G**) and the proliferation of non-naïve TCRαβ CD4 T cells, T_FH_ cells and T_REGS_ (% of KI-67^+^; **Figure 1H**) confirmed the lack of impact of DHODH inhibition on the CD4 T compartment. Collectively, the longitudinal immune monitoring of patients with RRMS showed that DHODH inhibition selectively impacts the CD8 T cell compartment.

### Teriflunomide Specifically Reduces the Proliferation and Immune Function of CD8 T Cells in Patients With RRMS

**Figure 2:**
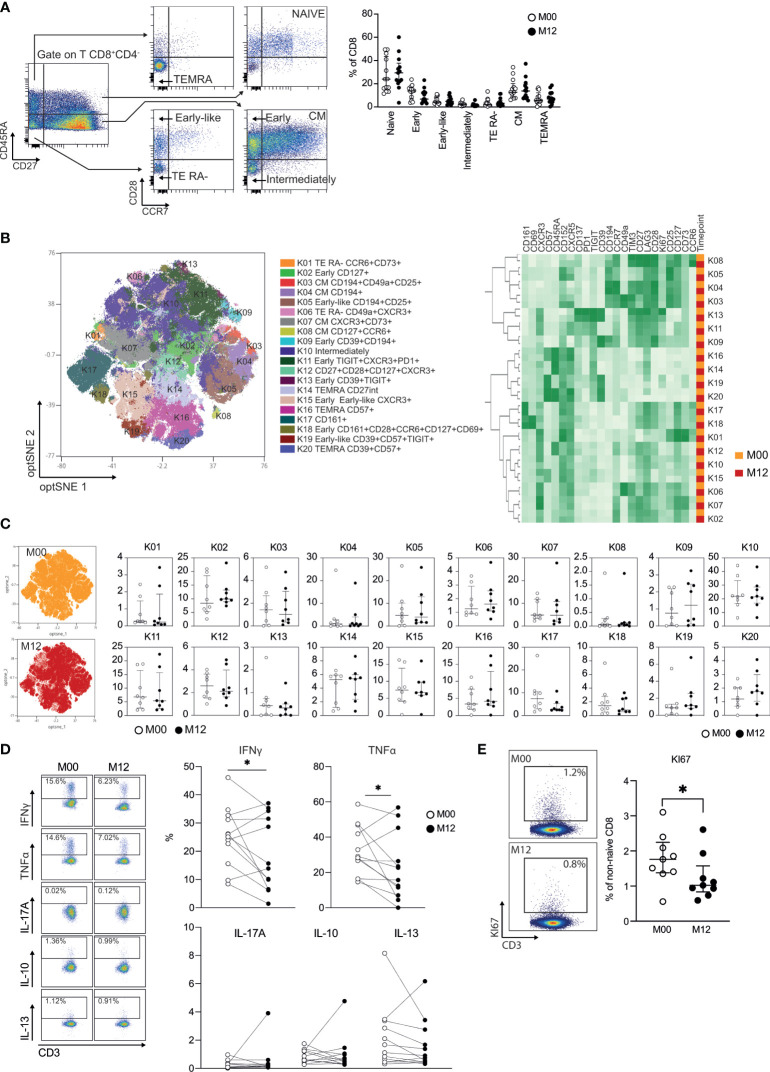
Teriflunomide specifically reduces the proliferation and immune function of CD8 T cells in patients with RRMS. **(A)** Frequency of CD8 T cell subsets in patients with RRMS at baseline (white) or 12 months post-teriflunomide treatment (black; n=13). The gating strategy is shown after initial gating of CD19^-^CD56^-^CD3^+^TCRγδ^-^CD8^+^CD4^-^ cells (TEMRA, CD45RA^+^CD27^-^CCR7^-^CD28^-^; NAÏVE, CD45RA^+^CD27^+^CCR7^+^CD28^+^; early-like, CD45RA^-^CD27^-^CCR7^-^CD28^+^; EM RA-, CD45RA^-^CD27^-^CCR7^-^CD28^-^; early, CD45RA^-^CD27^+^CCR7^-^CD28^+^; intermediate, CD45RA^-^CD27^+^CCR7^-^CD28^-^; CM, CD45RA^-^CD27^-^CCR7^+^CD28^+^). **(B)** optSNE projection of non-naïve αβ CD8 T cell clusters identified by FlowSOM metaclustering (left) and mean fluorescence intensity (MFI) as indicated (column-scaled z-scores) for the FlowSOM cluster (right). **(C)** Global optSNE projection of non-naïve αβ CD8 T cells for all participants pooled, with non-naïve αβ CD8 T cells of patients with RRMS at M00 (white) and M12 post-teriflunomide treatment (black) concatenated and overlaid. Percentage of non-naïve αβ CD8 T cells from each timepoint in each FlowSOM cluster. **(D)** T_H_1 cell (IFNγ, TNFα), T_H_2 cell (IL-13), T_REG_ (IL-10) and T_H_17 cell (IL-17) cytokine production by CD8 T cells of patients with RRMS at M00 (white) and M12 post-teriflunomide treatment (black) after polyclonal restimulation. **(E)** KI67 expression in non-naïve αβ CD8 T cells. **(A, C–E)** Each dot represents a unique patient, and the median and interquartile ranges (IQRs) are shown. Significance was determined by unpaired Mann-Whitney U or paired Wilcoxon test (*p < 0.05).

Seven major CD8 T cell populations were examined by using the combination of CD45RA, CD27, CCR7, and CD28 cell surface markers to define naïve (CD45RA^+^CD27^+^CCR7^+^CD28^+^), TEMRA (CD45RA^+^CD27^-^CCR7^-^CD28^-^), early-like effector memory (CD45RA^-^CD27^-^CCR7^-^CD28^+^), effector RA- [CD45RA^-^CD27^-^CCR7^-^CD28^-^ (TE RA^-^)], early memory [CD45RA^-^CD27^+^CCR7^-^CD28^+^ (early)], intermediate memory [CD45RA^-^CD27^+^CCR7^-^CD28^-^ (intermediate)], and central memory [CD45RA^-^CD27^+^CCR7^+^CD28^+^ (CM)] (**Figure 2A**) CD8 T cells [as described in a consensus paper (21)]. A nonsignificant increase in naïve CD8 T cells and a nonsignificant decrease in early CD8 T cells were observed in patients with RRMS 12 months posttreatment (**Figure 2A**). We then applied global high-dimensional mapping of the 27-parameter flow cytometry data to gain more insights into non-naïve αβ CD8 T cells. The opt-SNE representation of the data highlighted 20 populations that were identified by FlowSOM clustering and the comparison of marker expression (**Figure 2B**). The phenotype of the 20 populations at baseline was highly similar to those at 12 months posttreatment, as shown by the proximity of the 20 clusters after unsupervised clustering (**Figure 2B**). The opt-SNE representation of non-naïve CD8 T cells of patients at M00 was similar to that of patients at 12 months posttreatment (**Figure 2C**) and was shown by the analysis of the frequency of the 20 clusters of CD8 T cells (**Figure 2C**). Interestingly, the secretion of the proinflammatory cytokines IFNγ and TNFα produced by CD8 T cells was reduced in patients 12 months posttreatment (**Figure 2D**). The impact of DHODH inhibition was restricted to the production of proinflammatory cytokines by CD8 T cells, as the frequencies of IL-10 and IL-13 were similar before and 12 months posttreatment (**Figure 2D**). The reduced production of proinflammatory cytokines was confirmed in an analysis focused on TEMRA and EM CD8 T cell subsets (**Supplementary Figure 1**). Finally, a lower frequency of KI-67^+^ CD8 T cells was observed 12 months posttreatment (**Figure 2E**). The susceptibility of CD8 T cells of patients with RRMS to DHODH inhibition was confirmed *in vitro*, as the proliferation of EM and TEMRA CD8 T cells in response to TCR and IL-15 stimulation was blunted by teriflunomide, and the inhibition of proliferation can be reversed by added uridine (**Supplementary Figure 2**). In summary, we demonstrate that DHODH inhibition in patients with RRMS was associated with a lower *in vivo* proliferation of CD8 T cells with reduced proinflammatory properties 12 months after treatment.

### Teriflunomide Inhibits the Migration of EM and TEMRA CD8 T Cells of Patients With RRMS *In Vitro*

**Figure 3:**
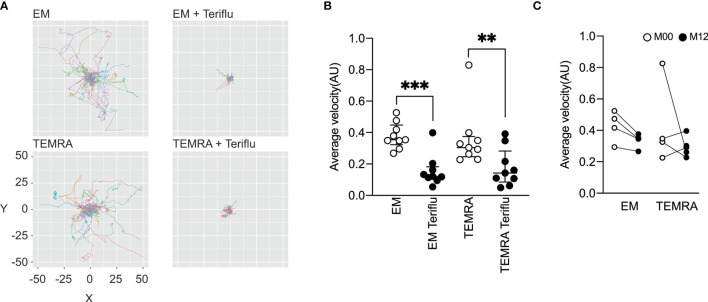
Teriflunomide inhibits the migration of EM and TEMRA CD8 T cells in patients with RRMS. **(A, B)** Migration of Fura-2-labeled CD8 T cell subsets of patients with RRMS onto TNFα (100 U/ml, O/N)-treated HDMEC monolayers and, as indicated, when teriflunomide (100 µM) was added 15’ after the initial recording. Paths of individual cells were aligned to their origins at x=y=0 **(A)**, and the average velocity **(B)** of CD8 T cell subsets is shown. **(C)** Migration of Fura-2-labeled CD8 T cell subsets of patients with RRMS at M00 (white) and at M12 (black) post-teriflunomide treatment onto TNFα (100 U/ml, O/N)-treated HDMEC monolayers. Average velocity of CD8 T cell subsets is shown. **(B, C)** Each dot represents a unique patient, and the median and interquartile ranges (IQRs) are shown. Significance was determined by unpaired Mann-Whitney U test (**p < 0.01; ***p < 0.001).

We next tested the impact of DHODH inhibition on the patrolling properties of EM and TEMRA CD8 T cells of with RRMS, two CD8 T cell subsets in which predominant T cell clones were notable among CNS lesions, CSF, and blood CD8^+^ T cells were found (15). The dynamic behavior of TEMRA and EM CD8 T cells on activated endothelial cells was tracked using live-cell imaging (**Figure 3**). This dynamic model of T cell interaction with the activated endothelium showed that the EM and TEMRA CD8 T cells of patients with RRMS exhibited similar patrolling behavior (**Figure 3**). Interestingly, the inhibition of DHODH resulted in the immediate and prolonged arrest of the EM and TEMRA CD8 T cells of patients with RRMS, as shown by the significant decrease in their average migratory velocity (**Figure 3B**). Finally, we assessed whether prolonged *in vivo* exposure to DHODH inhibition impacts the migratory properties of EM and TEMRA CD8 T cells of patients with RRMS. A nonsignificant decrease in the migratory velocity of the EM CD8 T cells was observed in the longitudinal follow-up of patients with RRMS 12 months after treatment (**Figure 3C**), whereas the velocity of the TEMRA CD8 T cells was not different before and 12 months posttreatment. Taken together, these results highlight that DHODH inhibition not only impacted the immune function of CD8 T cell subsets but also limited their migratory features.

### Metabolic Characterization of EM and TEMRA CD8 T Cells in Patients With RRMS

**Figure 4:**
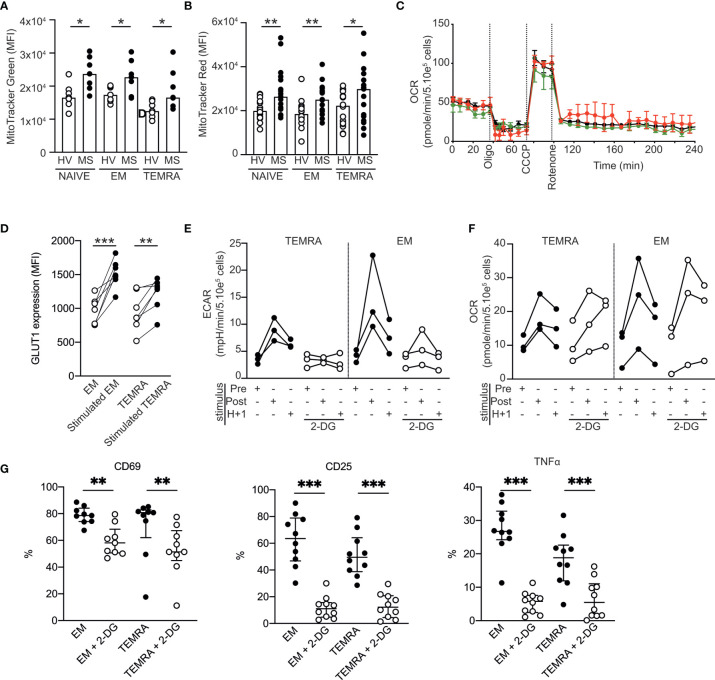
Metabolic characterization of CD8 T cell subsets in patients with RRMS. **(A, B)** Mitochondrial load **(A)** and mitochondrial membrane potential **(B)** of CD8 T cell subsets of HVs and patients with RRMS assessed according to the mean fluorescence intensity of MitoTracker green and red, respectively. **(C)** Oxygen consumption rate (OCR) of purified CD8 T cell subsets as measured by Seahorse technology before and after sequential addition of metabolic stress-inducing drugs. **(D)** Expression of GLUT-1 by EM and TEMRA CD8 T cells of patients with RRMS upon overnight stimulation with plate-bound anti-CD3. **(E, F)** Extracellular acidification rate [ECAR; **(E)**] and OCR **(F)** of EM and TEMRA CD8 T cells of patients with RRMS before and after polyclonal stimulation in the absence or in the presence of the glycolysis inhibitor 2-DG. **(G)** Expression of activation markers (CD69 and CD25) and proinflammatory cytokines (TNFα) in EM and TEMRA CD8 T cells of patients with RRMS after overnight stimulation with plate-bound anti-CD3 and IL-15 in the presence of medium control or 2DG. **(A, B, D–G)** Each dot represents a unique patient, and the median and interquartile ranges (IQRs) are shown. Significance was determined by unpaired Mann-Whitney U or paired Wilcoxon test (*p < 0.05; **p < 0.01; ***p < 0.001). **(C)** Representative OCR profile from one out of 5 patients with RRMS.

As DHODH has been shown to interfere with components of the electron transport chain, we wondered whether the high and selective susceptibility of CD8 T cells of patients with RRMS might be linked to impaired CD8 T cell energy metabolism. In the steady state, all CD8 T cell subsets (naïve, EM and TEMRA T cells) in patients with RRMS exhibited a higher mitochondrial mass than those of HVs (**Figure 4A**) with a higher energy activation in mitochondria, as shown by the mitochondrial membrane potential (MMP) measured using a voltage-dependent MitoTracker probe (**Figure 4B**). The absence of mitochondrial defects in CD8 T cell subsets in patients with RRMS was confirmed using a Seahorse assay. Basal respiration was similar across the different CD8 T cell subsets (**Figure 4C**), and as expected, the injection of oligomycin, an inhibitor of ATP synthase, induced a rapid decrease in the oxygen consumption rate (**Figure 4C**), demonstrating the mitochondrial coupling efficiency. Finally, the protonophore CCCP resulted in an increase in OCR in all CD8 T cell subsets (**Figure 4C**). Collectively, these results showed that CD8 T cell subsets of patients with RRMS exhibited well-functioning mitochondria under steady state conditions.

The ability to rapidly switch to glycolysis-based metabolism upon stimulation is a hallmark of memory CD8 T cells (23). We showed that, upon stimulation, EM and TEMRA CD8 T cell subsets of patients with RRMS exhibited modified nutrient uptake and metabolism to satisfy their bioenergetic needs, as shown by the increase in their ability to take up glucose (increased expression of glucose transporter 1 GLUT1; **Figure 4D**) and in the extracellular acidification rate (ECAR; an indicator of glycolysis; **Figure 4E**) and OCR within minutes following polyclonal stimulation (**Figure 4F**). As expected, the addition of 2-deoxyglucose (2-DG), which blocks the first step of the glycolytic pathway, prevented both the immediate and sustained increases in the ECAR while not increasing the OCR (**Figures 4E, F**). The impact of 2DG on glycolysis inhibition resulted in reduced activation of the EM and TEMRA CD8 T cell subsets of patients with RRMS, which showed lower expression of the early activation marker CD69 and the high-affinity IL-2 receptor CD25 and lower TNF α production (**Figure 4G**). Collectively, these data showed the ability of EM and TEMRA CD8 T cell subsets of patients with RRMS to adapt their metabolism to engage their effector function, and therefore, the selective susceptibility of CD8 T cells of patients with RRMS to DHODH inhibition was not related to impaired CD8 T cell energy metabolism.

## Discussion

Teriflunomide has been shown to be an effective therapy to treat patients with MS in placebo-controlled phase 3 trials [TEMSO (1) and TOWER (2)]. Recently, we confirmed these results in real-life settings, and we reported that teriflunomide and dimethyl fumarate have similar clinical effectiveness after 2 years of treatment of a large number of patients with RRMS (24). The underlying mechanism contributing to the efficacy of DHODH inhibition remains only partially elucidated. Here, using high-dimensional approaches, we demonstrate that patients with RRMS treated with teriflunomide exhibited a selective alteration of the CD8 T cell compartment as characterized by low homeostatic proliferation of CD8 T cells, low production of proinflammatory cytokines by memory CD8 T cells and decreased migratory velocity of memory CD8 T cells. Interestingly, B cell subsets and CD4 Treg, CD4 T_FH_ and CD4 T_H_ cell subsets were not impacted by 12 months of teriflunomide treatment.

It has been established that CD8 T cells play major roles in the pathogenesis of MS, as the clonal composition of T cells infiltrating active lesions shows predominant CD8 T cell infiltration (14, 15, 25–29). Expanded CD8 T cell clones have also been found in paired analysis of blood, cerebrospinal fluid (CSF) and active white matter lesions (14, 15, 27). CD8 T cells identified in white matter lesions were reported to show effector memory phenotypes (15, 27) with chronic activation (expressing CD95L and granzyme B) (27), with a high frequency of the CD8 T cells expressing coinhibitory receptors (TIM3 and PD1) (27) or a tissue-resident memory phenotype (29, 30). Indeed, single-cell RNA sequencing of cells infiltrating the cerebrospinal fluid (CSF) in patients with MS revealed that CD8 T cells were the main contributor of clonally expanded T cells with an activated tissue-resident memory phenotype (29), an observation in agreement with the *in situ* staining of CD8 T cells of white matter active lesions reported by Machado-Santos et al. (30) CNS-infiltrating CD8 T cell clones have also been detected in the CSF and in the blood, suggesting that uncommitted CD8 memory precursor T cells migrate from the periphery to the CSF and the CNS, where they acquire a tissue-resident memory phenotype. Alternatively, as shown for mouse epidermal resident memory CD8 T cells (31), naïve CD8 T cells can be primed for tissue residency. The selective alteration of CD8 T cell function reported herein strengthens previous findings showing that CD8 T cells are involved in MS and may partially explain the therapeutic efficacy of teriflunomide. An obvious limitation of our study is the small sample size. Nevertheless, we circumvented the confounding factor of treatment by systematically analyzing the effects of MS patient treatment through a longitudinal approach and by using paired analysis of samples (each patient being his own reference control).

Whether autoreactive T cells are recruited to the CNS remains a key unanswered question. Nevertheless, in this study, we showed that *in vitro* inhibition of DHODH alters the migratory properties of CD8 T cells in patients with RRMS, and interestingly, the velocity of CD8 T cell subsets of these patients was lower after 12 months of treatment with teriflunomide. These results suggest an additional mechanism of action of teriflunomide by which it limits the ability of memory CD8 T cells to infiltrate the CSF and CNS. This limitation of migratory fitness of memory CD8 T cells induced by teriflunomide has been previously suggested on the basis of the inhibition of integrin LFA-1 avidity after *in vitro* treatment with teriflunomide (32).

Teriflunomide has been shown to induce a modest but stable lymphopenia in patients with MS and the meta-analysis of patients enrolled in the TEMSO (1) and TOWER (2) phase 3 clinical trials show a mean decrease in leukocyte counts of 15% from baseline (4). Previous longitudinal studies have shown a stability in the composition of the CD4 and CD8 T subsets in patients RRMS analyzed 6 months after teriflunomide treatment (9, 10). Our findings confirm these results in MS patients after one-year of treatment with teriflunomide with no significant modification of the frequency of the CD4/CD8 T cell subsets analyzed with our comprehensive spectral flow cytometry panel. The current understanding of the mechanism of action of teriflunomide suggests that it can inhibit the proliferation of metabolically active T cells, a concept that has been further supported by the demonstration that high-affinity T cells are more susceptible to DHODH inhibition (10). No direct impact on cell survival has been reported (3). Based on surrogate markers of CD4 TH cells (i.e. chemokine receptors), a preferential reduction in TH1 CD4 T cells was reported while no modification in the other TH CD4 was observed including no impact on TH17 CD4 subsets (10). In the current study and based on the profiling of cytokines production by CD4 T cells, we also reported a trend in a lower secretion of TNFα and IFNγ by CD4 T cells after teriflunomide treatment (**Figure 1G**). We now also report that the *in vivo* proliferation of CD8 T cells and the production of pro-inflammatory cytokines by CD8 T cells are lower after treatment with teriflunomide. The impact of teriflunomide on CD8 T cells may suggest that, after treatment, MS patients may have a lower response upon viral infection. Nevertheless, published reports of large cohort of patients treated with teriflunomide failed to demonstrate any increase in opportunistic infections. The incidence of serious infections was not different between placebo-treated patients and Teriflunomide-treated patients (33) and the long-term follow-up of patients of the TEMSO studies did not evidence any serious opportunistic infections (33, 34). The ability to mount protective vaccine responses to recall antigens is preserved in MS patients treated with Teriflunomide (35). Finally, in the context of the current SARS-CoV-2 pandemic, case reports have shown that the continuation of Teriflunomide could be continued for MS patients and was not associated with negative outcome (36–42).

Although few reports have characterized immune metabolism in patients with autoimmune diseases such as systemic lupus erythematosus (43) or rheumatoid arthritis (44), the concept of a disease-specific signature of immune metabolism seems to prevail. In patients with MS, CD4 T cell metabolism has been shown to differ between patients with primary progressive MS (PPMS) and those with secondary progressive (SPMS) MS (45). Naïve and EM CD4 T cells of patients with PPMS have been shown to exhibit lower mitochondrial respiration, lower glycolytic rates and accumulation of disorganized mitochondria than CD4 T cells of SPMS patients (45). In contrast, oxidative phosphorylation and glycolysis in CD4 T cells have been found to be increased in patients with active RRMS compared to clinically stable patients and healthy controls (10). Here, we showed that CD8 T cell subsets (EM and TEMRA) exhibited an increased number of functional mitochondria in patients with RRMS compared to healthy controls. The fitness of the metabolic processes of EM and TEMRA CD8 T cells in patients with RRMS was confirmed by increased OCRs and ECARs in these cells upon stimulation and the ability of 2-DG treatment to blunt glycolysis and thus prevent an immune response. The upregulation of aerobic glycolysis upon pro-inflammatory stimulation is a shared characteristic seen in activated B cells, CD4 TH1/TH17 cells, CD8 T cells, educated NK cells and activated M1 macrophages. As a consequence, the interference of glycolysis is expected to control the pro-inflammatory response in patients, including in patients with MS. The selectivity of targeting metabolic processes is expected to be achieved by the high metabolic demands of target cells whereas other immune cells could be spared reducing unwanted side effects (46, 47).

Taken together, these findings strengthen the concept of disease-specific alteration of T cell immune metabolism in autoimmune diseases.

## Data Availability Statement

The raw data supporting the conclusions of this article will be made available by the authors, without undue reservation.

## Ethics Statement

The studies involving human participants were reviewed and approved by the University Hospital Committee and the Committee for the Protection of Patients from Biological Risks. The patients/participants provided their written informed consent to participate in this study.

## Author Contributions

GT: Methodology, Visualization, Validation, Formal analysis, Writing – Original draft. MC: Methodology, Formal analysis. AG: Resources, Data Curation, Formal analysis. JM: Resources. SW: Resources, Data Curation. CP, Methodology, Validation. SB: Conceptualization, Supervision, Writing – Review & Editing. DL: Conceptualization, Funding acquisition, Data Curation, Writing – Review & Editing. ND: Conceptualization, Funding acquisition, Software, Validation, Visualization, Formal analysis, Writing – Original draft, Writing – Review & Editing. All authors contributed to the article and approved the submitted version.

## Funding

This work was supported by a Genzyme Research grant and also partially supported by a grant from the LabEX IGO program supported by the National Research Agency *via* the “Investment into the Future” program (ANR-11-LABX-0016-01) and by the Public Investment Bank, also known as BPI France in the framework of the “Investment for the Future” programme (Programme d’Investissements d’Avenir). This work was also supported by the FP7 VISICORT project, which has received funding from the European Union’s Seventh Framework Programme for research, technological development and demonstration under grant agreement 602470.

## Conflict of Interest

DL has received consulting and lecturing fees, travel grants and unconditional research support from Biogen, Genzyme, Novartis, Merck, Roche, Sanofi, Medday, Teva Pharma and BMS. SW has received speaking honoraria and travel expense reimbursement for participation in scientific meetings and has participated in advisory boards in the past years with Alexion, Biogen, Merck, Novartis, Roche, Sanofi and Teva.

The remaining authors declare that the research was conducted in the absence of any commercial or financial relationships that could be construed as a potential conflict of interest.

## Publisher’s Note

All claims expressed in this article are solely those of the authors and do not necessarily represent those of their affiliated organizations, or those of the publisher, the editors and the reviewers. Any product that may be evaluated in this article, or claim that may be made by its manufacturer, is not guaranteed or endorsed by the publisher.

## Supplementary Material

Supplementary Figure 1 Hyporesponsiveness of EM and TEMRA CD8 T cells of teriflunomidetreated patients with RRMS. Production of IFNg and TNFa by CD8 EM and TEMRA cells of patients with RRMS at M00 (white) and at M12 (black) post-teriflunomide treatment. Each dot represents a unique patient, and the median and interquartile ranges (IQRs) are shown. Significance was determined by paired Wilcoxon test (**p < 0.01).

Supplementary Figure 2 Teriflunomide inhibits the proliferation of EM and TEMRA CD8 T cells of patients with RRMS. Percentage of CPDeFluor450low after stimulation of CD8 T cells of patients with RRMS with plate-bound anti-CD3 and IL15 in the presence of medium control or teriflunomide (100 µM) and, when indicated, uridine (50 µM). Each dot represents a unique patient, and the median is shown. Significance was determined by unpaired Mann-Whitney U test (**p < 0.01).

## Abbreviations

2-DG, 2-deoxyglucose (2-DG); CPD, cell proliferation dye eFluor450; DHODH, dihydroorotate dehydrogenase; ECAR, extracellular acidification rate; EM, effector memory; FlowSOM, flow cytometry self-organizing map; GLUT1, glucose transporter 1 GLUT1; MFI, mean fluorescence intensity; MMP, mitochondrial membrane potential; MS, multiple sclerosis; OCR, oxygen consumption rate; opt-SNE, optimized t-distributed stochastic neighbor embedding; PBMC, peripheral blood mononuclear cells; PPMS, primary progressive; TEMRA, CD45RA^+^ effector memory; T_FH_ cell, T follicular helper cell; T_H_ cell, T helper cell; T_REG_, regulatory T cell; SPMS, secondary progressive; RRMS, relapse-remitting MS.
