# Supplementary material for: Teriflunomide Treatment of Multiple Sclerosis Selectively Modulates CD8 Memory T Cells
Source: Front Immunol. 2021 Oct 5;12:730342. doi: 10.3389/fimmu.2021.730342 (PMC8552527; doi:10.3389/fimmu.2021.730342)
Supplement: Supplementary file 1 [file DataSheet_1.pdf]

Target	Fluorochrome	Clone Name	Provider	Assay[1]
CD3	BV421	UCHT1	BD Biosciences	1
CD8	VioGreen	BW135/80	Miltenyi	1
CD8	PE-Cy7	RPA-T8	BD Biosciences	1
CD45RA	APC-Vio770	TD611	Miltenyi	1
CD69	PerCp5.5	FN50	BD Biosciences	1
CD25	FITC	MA251	BD Biosciences	1
CD25	PE	BC96	Biolegend	1
CD3	BUV395	UCHT1	BD Biosciences	1
CD8	AF588	HIT8a	Biolegend	1
CCR7	BV605	G043H7	Biolegend	1
CD45RA	BV785	HI100	Biolegend	1
TNFα	PECy7	MAb11	Biolegend	1
Glut1	FITC	202915	R&D Systems	1
CD45RA	BUV496	HI100	BD Biosciences	2, 3
CXCR5	BUV563	RF8B2	BD Biosciences	2, 3
CCR4	BUV615	1G1	BD Biosciences	2
CD49a	BUV661	SR84	BD Biosciences	2
CD56	BUV737	NCAM16.2	BD Biosciences	2, 3
CD3	BUV805	OKT3	BD Biosciences	2, 3
PD-1	BV421	EH12.2H7	Biolegend	2
CD161	eFluor450	HP-3G10	Thermofisher	2
CD73	BV510	AD2	BD Biosciences	2
CD16	pacific orange	3G8	Thermofisher	2, 3
CD103	BV605	Ber-Act8	BD Biosciences	2
CD28	BV650	CD28.2	Biolegend	2
CCR6	BV711	G034E3	Biolegend	2
TCR Vα7.2	BV750	OF-5A12	BD Biosciences	2
4-1BB	BV786	4B4-1	BD Biosciences	2
CD39	BB515	TU66	BD Biosciences	2
CD57	FITC	HNK-1	Biolegend	2
CD8	spark blue550	SK1	Biolegend	2, 3
CD45	PerCP	2D1	Biolegend	2, 3
CD19	PerCp Cy 5.5	HIB19	Biolegend	2, 3
TCRγδ	PCPCeF710	B1.1	Thermofisher	2, 3
CD4	CF568	SK3	Cytek	2, 3
TIGIT	PE	A15153G	Biolegend	2
LAG-3	PE Dazzle 594	11C3C65	Biolegend	2
CTLA-4	PE Cy5	BNI3	BD Biosciences	2
CD25	PE Alexa F700	CD25-3G10	Thermofisher	2
CXCR3	PE Cy7	G025H7	Biolegend	2
CD27	APC	M-T271	Biolegend	2
TIM-3	Alexa 647	7D3	BD Biosciences	2
CD127	APC-R700	HIL-7R-M21	BD Biosciences	2

CD69	APC H7	FN50	Biolegend	2
CCR7	APC fire 810	G043H7	Biolegend	2, 3
IL-13	BV421	JES10-5A2	BD Biosciences	3
IL-10	BV711	JES3-9D7	BD Biosciences	3
TNFα	FITC	cA2	Miltenyi	3
IL-17a	PE	eBio64DEC17	eBiosciences	3

Supplementary Table 1. Listing of antibodies

[1] Conventional flow cytometry (1), Spectral cytometry – T cell Panel (2), Spectral cytometry – Functional Panel (3)
