# Supplementary material for: Teriflunomide Treatment of Multiple Sclerosis Selectively Modulates CD8 Memory T Cells
Source: Front Immunol. 2021 Oct 5;12:730342. doi: 10.3389/fimmu.2021.730342 (PMC8552527; doi:10.3389/fimmu.2021.730342)
Supplement: Supplementary file 2 [file DataSheet_2.pdf]

Reagent or Resource	Provider
Oligomycine	Sigma
CCCP	Sigma
Rotenone	Sigma
AntimycineA	Sigma
Seahorse XF buffer	Seahorse
2-Deoxy-D-glucose (2-DG)	Sigma
Teriflunomide	Sigma
Uridine	Sigma
PMA	Sigma
Calcium Ionophore	Sigma
BrefeldineA	Sigma
Cell Proliferation Dye V450	eBiosciences
Human TNFα	R&D Systems
Human IL-15	Miltenyi
human REAlease CD8 MicroBead Kit	Miltenyi
TexMacs Medium	Miltenyi
Endothelial Cell Growth Medium	LONZA
Fura-2	Thermofisher
e-Biosciences CytoFix/Perm kit	Thermofisher
μ-Slide 8 wells	Ibidi
MitoTracker Red	Thermofisher
MitoTracker Green	Thermofisher
CTL Wash supplement 10X	Immunospot
Benzonase	Sigma
Brillant Stain Buffer Plus	BD
Live/dead Fixable Blue for UV excitation	ThermoFischer

Supplementary Table 2. Listing of reagents
