# Supplementary figures and images for: Teriflunomide Treatment of Multiple Sclerosis Selectively Modulates CD8 Memory T Cells
Source: Front Immunol. 2021 Oct 5;12:730342. doi: 10.3389/fimmu.2021.730342 (PMC8552527; doi:10.3389/fimmu.2021.730342)

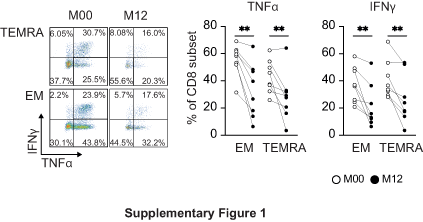

Supplement: Supplementary Figure 1 — Hyporesponsiveness of EM and TEMRA CD8 T cells of teriflunomidetreated patients with RRMS. Production of IFNg and TNFa by CD8 EM and TEMRA cells of patients with RRMS at M00 (white) and at M12 (black) post-teriflunomide treatment. Each dot represents a unique patient, and the median and interquartile ranges (IQRs) are shown. Significance was determined by paired Wilcoxon test (**p < 0.01). [file Image_1.tif]

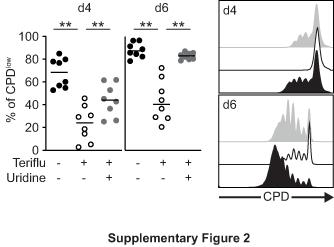

Supplement: Supplementary Figure 2 — Teriflunomide inhibits the proliferation of EM and TEMRA CD8 T cells of patients with RRMS. Percentage of CPDeFluor450low after stimulation of CD8 T cells of patients with RRMS with plate-bound anti-CD3 and IL15 in the presence of medium control or teriflunomide (100 µM) and, when indicated, uridine (50 µM). Each dot represents a unique patient, and the median is shown. Significance was determined by unpaired Mann-Whitney U test (**p < 0.01). [file Image_2.tif]
